# Editorial Comment: Objective Assessment and Standard Setting for Basic Flexible Ureterorenoscopy Skills Among Urology Trainees Using Simulation-Based Methods

**DOI:** 10.1590/S1677-5538.IBJU.2021.02.07

**Published:** 2021-02-03

**Authors:** Alexandre Danilovic

**Affiliations:** 1 Hospital das Clínicas da Faculdade de Medicina da USP São Paulo SP Brasil Serviço de Urologia, Hospital das Clínicas da Faculdade de Medicina da USP - HCFMUSP, São Paulo, SP, Brasil

Mitchell Goldenberg ^1^, Michael Ordon ^1^, John R D'A Honey ^1^, Sero Andonian ^2^, Jason Y Lee ^3^

## COMMENT

Flexible ureterorenoscopy (fURS) is a frequent procedure in urology practice (1). Graduating residents are expected to be competent in the skills necessary to perform fURS.

This study aim was to assess the basic fURS skills of graduating residents using a procedure––specific ureteroscopic global rating scale. Forty urology residents in their final year of residency from eleven programs in Canada were evaluated while performing a standardized task using a fURS model. More than 79% of then reported experience in more than 50 fURS. The task was to inspect the entire collecting system and to relocate two lower caliceal stones to an upper calyx using a tipless basket. Expert surgeons and anonymous people from a web platform (crowd-workers) rated the performances. Before the task, all residents felt they would be competent in fURS. However, only 56.9% of the residents were competent enough to perform fURS based on expert ratings and 61.4% based on crowd-workers ratings. Poor outcomes of surgical procedures may be caused by several reasons but surgeon inability must not be one of them (2).

The COVID-19 pandemic caused an abrupt decrease in surgical volume jeopardizing urology training (3, 4). Simulators and training models already exist to improve proficiency in most common endoscopic procedures and should be more used now (5, 6). While every effort should be made to mitigate clinical and surgical losses caused by the pandemic, residents should be evaluated in an individual basis to identify and to fix possible gaps before the end of their professional training.
